# *Cardinal*: an R package for statistical analysis of mass spectrometry-based imaging experiments

**DOI:** 10.1093/bioinformatics/btv146

**Published:** 2015-03-15

**Authors:** Kyle D. Bemis, April Harry, Livia S. Eberlin, Christina Ferreira, Stephanie M. van de Ven, Parag Mallick, Mark Stolowitz, Olga Vitek

**Affiliations:** ^1^Department of Statistics and; ^2^Department of Chemistry, Purdue University, West Lafayette, IN 47907 USA,; ^3^Canary Center at Stanford for Cancer Early Detection, Stanford University School of Medicine, Palo Alto, CA 94304 USA,; ^4^College of Science and; ^5^College of Computer and Information Science, Northeastern University, Boston, MA 02115 USA

## Abstract

*Cardinal* is an R package for statistical analysis of mass spectrometry-based imaging (MSI) experiments of biological samples such as tissues. *Cardinal* supports both Matrix-Assisted Laser Desorption/Ionization (MALDI) and Desorption Electrospray Ionization-based MSI workflows, and experiments with multiple tissues and complex designs. The main analytical functionalities include (1) image segmentation, which partitions a tissue into regions of homogeneous chemical composition, selects the number of segments and the subset of informative ions, and characterizes the associated uncertainty and (2) image classification, which assigns locations on the tissue to pre-defined classes, selects the subset of informative ions, and estimates the resulting classification error by (cross-) validation. The statistical methods are based on mixture modeling and regularization.

**Contact**: o.vitek@neu.edu

**Availability and implementation**: The code, the documentation, and examples are available open-source at www.cardinalmsi.org under the Artistic-2.0 license. The package is available at www.bioconductor.org.

## 1 Introduction

Mass spectrometry-based imaging (MSI) experiments characterize the chemical composition of biological samples (such as tissues) or non-biological samples at spatial resolution (Watrous *et al.*, 2011). The experiments repeatedly acquire mass spectra at gridded locations on a tissue. Two typical goals of statistical analysis of MSI are *image segmentation*, which partitions a tissue into regions of homogeneous spectral profiles, and *image classification*, which assigns locations of the tissue to pre-defined classes based on their spectral profiles. However, achieving these goals is often quite difficult due to the large and complex nature of the datasets, and due to the biological and technical variation in intensities of spectral features. Statistical inference is key for distinguishing the systematic signals in the spectra from noise. The availability of statistical methods and software for MSI experiments is currently limited.

We introduce *Cardinal*, an open-source R-based software package for processing and visualization of mass spectra, and for statistical segmentation and classification of the resulting images. *Cardinal* differs from other publicly available software tools such as BioMap, DataCube Explorer and MSiReader in its emphasis on statistical modeling and inference. It differs from commercial tools such as SCiLS Lab (SCiLS), flexImaging (Bruker), HDI (Waters) and TissueView (AB Sciex) in being open-source. It differs from the existing R packages for mass spectrometry such as MALDIquant and MSnbase in being designed specifically for MSI.

## 2 Description

### 2.1 Applicability and requirements

*Cardinal* is applicable to experiments aiming at segmentation and classification of a single tissue, or multiple tissues collected across biological subjects. It is applicable to Desorption Electrospray Ionization workflows, and to Matrix-Assisted Laser Desorption/Ionization (MALDI) workflows analyzing either intact or *in* *situ*-digested proteins. *Cardinal* has been tested on raw MS1 spectra from Thermo LTQ linear ion trap, ABSciex TOF/TOF and Bruker Autoflex MALDI-TOF instruments with resolving powers ranging from 1000 to 22 000. *Cardinal* is compatible with Windows, Mac and Linux operating systems. The size of the input dataset must be such that it can be loaded entirely into computer memory. *Cardinal* runs optimally when the available memory is twice the size of the dataset.

### 2.2 Data import, processing and visualization

*Cardinal* supports input data in the imzML format (Schramm *et al.*, 2012), and the Analyze7.5 format. Free converters to imzML are available for most other formats at www.imzml.org, and the converted imzML input data can be read into *Cardinal*.

*Cardinal* implements a complete set of common spectral processing methods (Yang *et al.*, 2009), including normalization (e.g. using total ion current), baseline correction (e.g. using median interpolation), peak detection [e.g. using LIMPIC (Mantini *et al.*, 2007)] and peak alignment (e.g. using mean spectrum).

*Cardinal* visualizes mass spectra, molecular ion images and results of the statistical analyses. The images are optimized with contrast enhancement and smoothing. The plots can be conditioned on experimental metadata (such as the type of the tissue), and viewed separately using a grid layout with multiple conditions, or jointly in a superposition.

### 2.3 Functionalities for statistical analysis

**Fig. 1.**
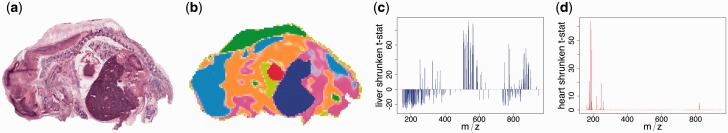
Unsupervised model-based segmentation of a cross-section of a pig fetus. (**a**) Optical image of a hematoxylin & eosin-stained tissue highlights its morphology, e.g. the brain (left), the heart (center) and the liver (dark region below the heart). (**b**) Joint segmentation of five adjacent tissue sections from 28 016 non-background pixels and 10 200 mass features. *Cardinal* detected 298 peaks during peak picking. The segmentation with Spatial Shrunken Centroids and Spatially Aware distance selected 11 tissue segments. (**c**) The *t*-statistics quantified the relative importance of the peaks in the liver. Ninety-two peaks were systematically enriched and 153 were systematically absent, as compared with the mean spectrum. (**d**) As in (c), but for the heart segment. Only 23 peaks were systematically enriched in the heart, and none were systematically absent. Similar analyses can be performed in a supervised manner for image classification

For image segmentation, *Cardinal* implements several existing methods, e.g. principle component analysis and Spatially-Aware (SA) and Spatially Aware Structurally Adaptive (SASA) segmentation (Alexandrov and Kobarg, 2011). *Cardinal* also introduces a novel method, called Spatial Shrunken Centroids, for model-based unsupervised image segmentation (Fig. 1). It combines the spatial distance from SA and SASA with the mixture modeling and regularization from Nearest Shrunken Centroids (Tibshirani *et al.*, 2003). The mixture modeling allows us to estimate the probability that a location on the tissue belongs to a particular segment. Statistical regularization allows us to automatically select the spectral features that define each segments, as well as the total number of segments.

For image classification, *Cardinal* implements partial least squares discriminant analysis and orthogonal projections to latent structures discriminant analysis (Dill *et al.*, 2010). *Cardinal* also introduces a novel Spatial Shrunken Centroids for model-based image classification, which utilizes the same principles as the model-based image segmentation but works in a supervised manner. For all the methods, *Cardinal* automates the estimation of classification error rate by (cross-)validation.

### 2.4 Implementation and performance

*Cardinal* employs efficient data structures to store the data and the metadata, and optimized methods for data manipulation. As the result, *Cardinal* can be used with any dataset that fits in the computer memory. For example, the dataset in Figure 1 with 28 016 pixels was 2.2 GB before the peak picking, and the processed version was 63.7 MB after the peak picking. Computation of the first 20 principal components took 86.9 sec on the raw data and 4.3 s on the picked peaks on a MacBook Pro with a 2.6 GHz Intel Core i7 and 16 GB memory. The segmentation with Spatial Shrunken Centroids on the picked peaks took 241 s (shortest) to 827 s (longest), depending on the initial values of regularization parameters and the number of clusters, on the same computer.

*Cardinal* facilitates the development of new functionalities, and the interoperability with other software. For example, raw mass spectra can be stored as either a R matrix or any matrix-like object, such as a sparse matrix. Most of the processing methods use an extendable framework pixelApply, similar to the apply family of methods in R. The ResultSet data structure allows the developers to store the results of any analyses, and directly access the *Cardinal*’s plotting capabilities. *Cardinal* also has functions for simulating mass spectra, to assist method testing. It is publicly available at www.bioconductor.org

## 3 Conclusions

*Cardinal* is a general, flexible, open-source tool for the analysis of MSI experiments. It can be used by researchers with and without background in R and computing. For experimenters, *Cardinal* provides a full toolchain for multiple workflows, with emphasis on multivariate statistical modeling, inference and model-based visualization. For developers, *Cardinal* provides a foundation for designing and implementing new methods of computational and statistical analysis of MSI experiments. Users can find more support through the Google Group, accessible through the project website.
